# Long‐term predictive value of stroke volume index obtained from right heart catheterization: Insights from the veterans affairs clinical assessment, reporting, and tracking program

**DOI:** 10.1002/clc.23418

**Published:** 2020-07-30

**Authors:** Anthony A. Bavry, Edward Hess, Stephen W Waldo, Anna E. Barón, Dharam J. Kumbhani, Deepak L. Bhatt, Ambarish Pandey

**Affiliations:** ^1^ Department of Medicine University of Texas Southwestern Dallas Texas USA; ^2^ Rocky Mountain Regional VA Medical Center Denver Colorado USA; ^3^ University of Colorado School of Medicine Denver Colorado USA; ^4^ Department of Biostatistics and Informatics, Colorado School of Public Health University of Colorado Anschutz Medical Campus Aurora Colorado USA; ^5^ Brigham and Women's Hospital Boston Massachusetts USA

**Keywords:** cardiac catheterization/diagnostic interventional, cardiac function, cardiomyopathy, heart failure, chronic < ischemic heart disease

## Abstract

**Background:**

Right heart catheterization‐derived hemodynamic parameters have been associated with short‐term prognosis.

**Hypothesis:**

Hemodynamic parameters will be associated with long‐term prognosis.

**Methods:**

Retrospective cohort study from the Veterans Affairs Clinical Assessment, Reporting, and Tracking Program included patients who underwent an index right heart catheterization between 2008 and 2016. Cox proportional hazard models were used to examine the association between stroke volume index and all‐cause mortality.

**Results:**

For the final cohort of 37 209 patients, mean follow‐up was 3.7 ± 2.5 years. All‐cause mortality was 42.0% in the low (<35 cc/beat/m^2^) compared with 33.2% in the normal stroke volume index group (≥35 cc/beat/m^2^). In adjusted analysis, low stroke volume was significantly associated with higher mortality risk (HR (95% CI) 1.14 (1.10‐1.18); *P* < .001) independent of clinical parameters. The area under the curve (AUC) for continuous measures of stroke volume index at predicting mortality in a Cox proportional hazard model was 0.56 at 3 years. When stroke volume index was combined with 14 clinical covariates, the AUC was 0.70 at 3 years. The addition of stroke volume index to these clinical covariates did not increase the discriminatory ability of the model at 1 year in a clinically meaningful way (integrated discrimination improvement index = 0.0021, 95% CI: 0.0010‐0.0034).

**Conclusions:**

The long‐term prognostic value of right heart catheterization‐derived stroke volume index appears to be marginal. While there was a weak association of low stroke volume index and excess mortality, inclusion of this parameter to a set of clinical covariates did not improve prognostic discrimination.

## 1 INTRODUCTION

Hemodynamic parameters obtained from right heart catheterization, such as cardiac output, provide short‐term prognostic information in patients with cardiogenic shock.^1^, ^2^, ^3^ Similarly, among patients with valvular disease such as aortic stenosis, echocardiography‐derived stroke volume index has been associated with long‐term prognosis with a significantly higher risk of mortality noted among those with lower stroke volume.^4^, ^5^, ^6^ Despite the perceived prognostic utility of invasive and noninvasive measures of cardiac performance, the Evaluation Study of Congestive Heart Failure and Pulmonary Artery Catheterization Effectiveness trial failed to demonstrate any improvements in short‐term mortality with routine invasive hemodynamic assessment using right heart catheterization compared with conventional clinical management among patients with decompensated heart failure without cardiogenic shock.^7^ Accordingly, the current American College of Cardiology/American Heart Association guidelines recommend use of invasive hemodynamic assessment with pulmonary artery catheters only in patients with cardiogenic shock or mechanical ventilation, but not for routine management of heart failure.^8^ Recent studies have demonstrated an increase in the use of pulmonary artery catheters in patients with heart failure and valvular heart disease; however, the prognostic utility of hemodynamic parameters from right heart catheterization to predict long‐term risk is unknown.^9^, ^10^ In this study, our aim was to determine if right heart catheterization‐derived stroke volume index can improve long‐term prognostication compared with readily available clinical variables among a broad cohort of patients. We hypothesized that right heart catheterization‐derived stroke volume index, would significantly improve the risk prediction for long‐term mortality above and beyond clinical risk factors.

## 2 METHODS

### 2.1 Study cohort and data source

The present study used data from the Veterans Affairs Clinical Assessment, Reporting, and Tracking (VA CART) Program, a national quality and safety program for invasive cardiac procedures in the VA Healthcare System. The study was conducted according to high ethical standards. Currently, the Program uses an internally developed software application within the VA electronic health record for documentation of all cardiac catheterization procedures.^11^ Patient and procedural data on cardiac catheterizations are linked to the VA electronic health record. Quality checks are performed regularly to ensure completeness and accuracy. Additional details have been previously described.^12^ The study cohort consisted of all index right heart catheterizations with or without left heart catheterizations that were performed in the VA Health System from October 1, 2008 to September 30, 2016. Patients were excluded if they were missing left ventricular ejection fraction, systolic systemic blood pressure, mean pulmonary artery pressure, body mass index, or any of the hemodynamic measurements of interest (see below). For patients who underwent multiple right heart catheterizations during the study period, only the index procedure (ie, earliest qualifying study) was included for analysis. As a sensitivity analysis, a landmark analysis was performed on patients alive 30 days following the index procedure.

### 2.2 Study variables

Demographic variables (age, race, etc.) were extracted from the administrative database. History of aortic valve replacement (surgical or transcatheter) within the previous 12 months was obtained from current procedural terminology codes. This time period was selected to minimize the likelihood for aortic prosthesis dysfunction. Laboratory values were obtained within 6 months prior to the right heart catheterization procedure. Systemic blood pressure was obtained from central aortic pressure, or from noninvasive blood pressure if the former was not available. Cardiac output was preferentially obtained from thermodilution method, or the Fick method if the former was not available.^11^ Stroke volume was defined as cardiac output divided by heart rate. Cardiac power output was defined as (mean arterial blood pressure × cardiac output)/451. Stroke work was defined as (mean arterial blood pressure ‐ pulmonary capillary wedge pressure) × (0.0136 × stroke volume). Left ventricular work was defined as (mean arterial blood pressure − pulmonary capillary wedge pressure) × 0.0136 × cardiac output. Each of the hemodynamic parameters was divided by body surface area to obtain its indexed values. Biologically implausible values were excluded.

### 2.3 Statistical analysis

The baseline characteristics of the study cohort were summarized by median (with interquartile interval) for continuous variables and as percentage (frequency) for categorical variables. The unadjusted and adjusted association between invasive measures of cardiac performance and the risk of long‐term mortality were assessed using Cox proportional hazards models which included a random intercept for site (ie, a frailty model) using the *coxph* function from the survival package in R. The proportional hazards assumption was assessed for covariates in the model using the *cox.zph* function, and no major violations were observed. Separate models were constructed for each invasive measure of cardiac performance as the primary exposure variable. Each measure was modeled as a continuous variable (a restricted cubic spline with four degrees of freedom) when assessing its utility as a predictor (eg, when calculating metrics such as area under the curve [AUC]). Additionally, categorical versions of these measures (split at the 20th, 40th, 60th, and 80th percentiles with 40th to 60th percentile as the referent) were modeled to provide easily interpretable estimates of the effect of each predictor across the range of possible predictor values. The covariates in the adjusted models included the following biologically relevant potential confounders: age, body mass index, left ventricular ejection fraction, mean pulmonary artery pressure, systemic systolic blood pressure, prior myocardial infarction, prior stroke/transient ischemic attack, heart failure, cancer, chronic obstructive pulmonary disease, cirrhosis, estimated glomerular filtration rate, serum sodium, and hemoglobin.^13^, ^14^ Separate models were also constructed with categorical measures of stroke volume index derived using clinically meaningful thresholds (ie, <35 cc/beat/m^2^ vs ≥35 cc/beat/m^2^). The predictive performance of models with vs without invasive measures of cardiac performance for the risk of mortality 1‐, 2‐, and 3‐years following the procedure were assessed by calculating the AUC for the receiver operating characteristic. Additionally, the integrated discrimination improvement metric was calculated at 1 year based on the method of Chambless et al using a clustered bootstrap.^15^, ^16^ Data preparation and statistical analyses were performed using SAS version 9.4 and R version 3.6.1.

## 3 RESULTS

The final study cohort consisted of 37 209 patients. The median age was 66.5 years and the most prevalent comorbidity was hypertension, which was present in 88.6%. Approximately 40% had undergone percutaneous or surgical revascularization (Table 1). The median stroke volume index was 34.6 cc/beat/m^2^. Complete hemodynamic parameters are provided in Table 2. Over a mean follow up of 3.7 years (±2.5 years), 14 034 patients (37.7%) died in our study cohort.

**TABLE 1:** Baseline characteristics of the study participants

Baseline characteristics	Total cohort (n = 37 209)	SVi <35 cc/m^2^/beat (n = 19 097)	SVi ≥35 cc/m^2^/beat (n = 18 112)	*P*‐value
Age, median years (IQR)	66.5 (61.2, 73.6)	66.5 (61.1, 74.0)	66.5 (61.3, 73.2)	.14
Male, % (n)	96.8% (36 025)	97.0 (18 519)	96.7 (17 506)	.08
BMI, median kg/m^2^ (IQR)	29.6 (25.8, 34.3)	29.3 (25.6, 34.0)	29.9 (26.1, 34.7)	<.001
Race, % (n)				<.001
White	79.8 (29 680)	77.5 (14 805)	82.1 (14 875)	
African American	18.2 (6769)	20.4 (3889)	15.9 (2880)	
LVEF, median % (IQR)	54.0 (35.0, 60.0)	45.0 (30.0, 57.5)	55.0 (45.0, 62.5)	<.001
*Comorbidities, % (n)*				
Hypertension	88.6 (32 973)	88.6 (16 924)	88.6 (16 049)	.97
Heart failure	59.3 (22 060)	70.6 (13 480)	47.4 (8580)	<.001
Diabetes	47.9 (17 824)	49.0 (9361)	46.7 (8463)	<.001
COPD	33.4 (12 416)	35.9 (6851)	30.7 (5565)	<.001
Chronic kidney disease	31.3 (11 653)	32.5 (6200)	30.1 (5453)	<.001
Dialysis	4.7 (1744)	3.3 (630)	6.2 (1113)	<.001
Atrial fibrillation	28.9 (10 748)	36.3 (6923)	21.1 (3825)	<.001
Prior MI	28.5 (10 607)	30.6 (5846)	26.3 (4761)	<.001
PAD	20.6 (7668)	21.1 (4035)	20.1 (3633)	.011
Prior PCI	20.5 (7623)	21.0 (4013)	19.9 (3610)	.01
Prior CABG	19.4 (7220)	20.2 (3857)	18.6 (3363)	<.001
Cancer	14.5 (5387)	14.2 (2709)	14.8 (2678)	.10
Prior stroke/TIA	8.6 (3198)	9.2 (1766)	7.9 (1432)	<.001
Cirrhosis	7.5 (2776)	6.1 (1164)	8.9 (1612)	<.001
*Laboratory analysis, mg/dL*				
Serum sodium	139 (137, 141)	139 (136, 141)	139.0 (137.0, 141.0)	<.001
Hemoglobin	13.0 (11.5, 14.3)	13.2 (11.8, 14.5)	12.8 (11.3, 14.1)	<.001
Serum creatinine	1.1 (0.9, 1.4)	1.1 (1.0, 1.4)	1.1 (0.9, 1.4)	<.001

Abbreviations: BMI, body mass index; CABG, coronary artery bypass grafting; COPD, chronic obstructive pulmonary disease; IQR, interquartile range; MI, myocardial infarction; PAD, peripheral arterial disease; PCI, percutaneous coronary intervention; TIA, transient ischemic attack.

**TABLE 2:** Cardiac catheterization pressures and hemodynamic parameters among study participants

Hemodynamic parameters	Median (interquartile range)
Systolic blood pressure, mm Hg	130.3 (120.2, 140.0)
Diastolic blood pressure, mm Hg	73.5 (67.3, 80.0)
Mean arterial blood pressure, mmHg	92.5 (86.0, 99.0)
Pulmonary capillary wedge pressure, mmHg	16.0 (11.0, 23.0)
Pulmonary systolic artery pressure, mm Hg	40.0 (32.0, 53.0)
Pulmonary diastolic artery pressure, mm Hg	18.0 (13.0, 25.0)
Mean pulmonary artery pressure, mm Hg	27.0 (20.0, 36.0)
Right atrial pressure, mm Hg	9.0 (5.0, 13.0)
Stroke volume, cc/beat	72.9 (56.7, 90.7)
Stroke volume index, cc/beat/m^2^	34.6 (27.5, 42.5)
Cardiac output, L/min	5.1 (4.1, 6.1)
Cardiac index, L/min/m^2^	2.4 (2.0, 2.9)
Cardiac power output, W	1.0 (0.8, 1.3)
Cardiac power output index, W/m^2^	0.5 (0.4, 0.6)
Stroke work, g·m	75.3 (55.1, 96.8)
Stroke work index, g·m/m^2^	35.8 (26.5, 45.5)
Left ventricular work, kg·m/min	5.2 (4.0, 6.5)
Left ventricular work index, kg·m/min/m^2^	2.5 (1.9, 3.1)

### 3.1 Association between stroke volume index and risk of mortality

**FIGURE 1:**
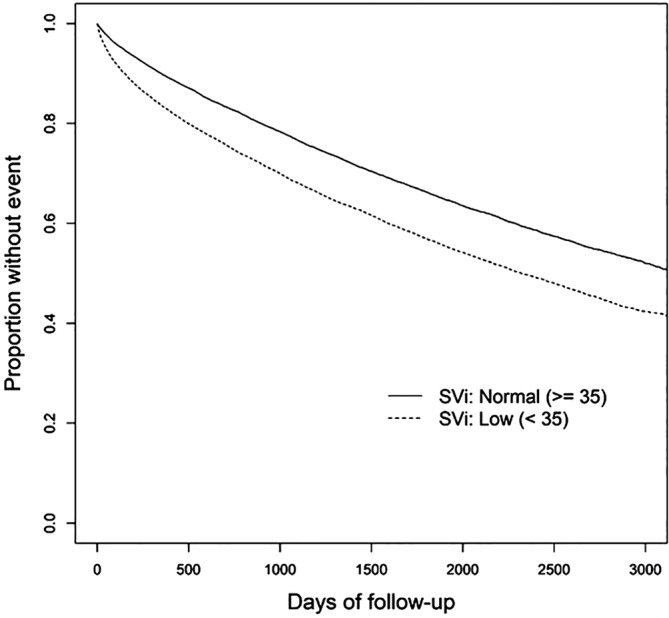
Survival analysis for all‐cause mortality among those with low stroke volume index vs normal stroke volume index

In unadjusted analysis, lower stroke volume index was associated with higher risk of mortality. Compared with the third (middle) quintile of stroke volume index, the risk of mortality was 54% higher in individuals in the lowest quintile and 9% lower in those in the highest quintile of stroke volume index. The association between higher stroke volume index and lower risk of mortality attenuated slightly but remained statistically significant in adjusted analysis such that individuals in the lowest and highest quintile of stroke volume index had 18% higher and 7% lower risk of mortality, respectively, as compared with the participants in third quintile of stroke volume index (Table 3). In time to event analysis using stroke volume index as a dichotomous variable (low vs high) based on the clinically meaningful cut off of 35 mL/m^2^, low stroke volume index was associated with higher risk of all‐cause mortality in both unadjusted (HR (95% CI): 1.39 (1.34‐1.44), *P* < .001, Figure 1) and adjusted analysis (HR (95% CI): 1.14 (1.10 to 1.18), *P* < .001).

**TABLE 3:** Unadjusted and adjusted association of stroke volume index and all‐cause mortality

Stroke volume index	HR	95% CI	*P*‐value
*0‐20th percentile (3.45, 25.7)*			
Unadjusted	1.54	1.46‐1.62	<.001
Adjusted	1.18	1.12‐1.25	<.001
*20‐40th percentile (25.71, 31.9)*			
Unadjusted	1.25	1.19‐1.32	<.001
Adjusted	1.10	1.04‐1.16	<.001
*40‐60th percentile (31.9, 37.5)*	1	—	—
*60‐80th percentile (37.50, 44.6)*			
Unadjusted	0.91	0.87‐0.97	.001
Adjusted	0.96	0.91‐1.01	0.15
*80‐100th percentile (44.59, 112.4)*			
Unadjusted	0.91	0.86‐0.96	.001
Adjusted	0.93	0.88‐0.98	.008

*Note:* Bracket is inclusive of that value, while parenthesis is exclusive of that value. Cox proportional model adjusted for age, body mass index, left ventricular ejection fraction, mean pulmonary artery pressure, systolic blood pressure, prior myocardial infarction, prior stroke or transient ischemic attack, congestive heart failure, cancer, chronic obstructive pulmonary disease, cirrhosis, estimated glomerular filtration rate, serum sodium, and hemoglobin.

Among other measures of cardiac performance, the association of stroke work index and risk of mortality was linear and similar to stroke volume index, with significantly higher risk of mortality in the lowest quintile and a graded decline in risk across increasing quintiles. In contrast, for cardiac index, cardiac power index, and left ventricular work index, the associations with mortality were non‐linear, with a significantly higher risk in the lowest quintile but not for other data derived groups (vs quintile 3, reference) in fully adjusted model as shown in Table 4.

**TABLE 4:** Adjusted association of other invasive measures of cardiac performance and all‐cause mortality

Cardiac performance parameter	HR (95% CI)	*P*
*Cardiac index, L/m* ^*2*^ *(ref: Q3)*		
Q1 [0.57, 1.91)	1.13 (1.07‐1.19)	<.001
Q2 [1.91, 2.24)	1.04 (0.99‐1.10)	.13
Q4 [2.56, 2.99)	0.99 (0.94‐1.05)	.75
Q5 [2.99, 7.47]	1.01 (0.96‐1.07)	.65
*Cardiac power index, W/m* ^*2*^ *(ref: Q3)*		
Q1 [0.11, 0.38)	1.15 (1.09‐1.21)	<.001
Q2 [0.38, 0.46)	1.04 (0.99–1.10)	.12
Q4 [0.53, 0.62)	1.00 (0.95‐1.06)	.95
Q5 [0.62, 1.59]	1.00 (0.95‐1.07)	.87
*Stroke work index, g/m/beat/m* ^*2*^ *(ref: Q3)*		
Q1 [1.9, 24.7)	1.09 (1.03‐1.15)	.003
Q2 [24.7, 32.5)	1.05 (1.00‐1.11)	.064
Q4 [39.6, 48.3)	0.94 (0.89‐1.00)	.033
Q5 [48.3, 125.3]	0.89 (0.84‐0.94)	<.001
*Left ventricular work index, g.m/m* ^*2*^ *(ref: Q3)*		
Q1 [0.18, 1.82)	1.11 (1.05‐1.17)	<.001
Q2 [1.836, 2.28)	1.06 (1.00–1.11)	.048
Q4 [2.696, 3.24)	1.05 (0.99‐1.11)	.10
Q5 [3.23, 8.60]	1.04 (0.98‐1.10)	.25

*Note:* Bracket is inclusive of that value, while parenthesis is exclusive of that value. Cox proportional hazards model adjusted for age, body mass index, left ventricular ejection fraction, mean pulmonary artery pressure, systolic blood pressure, prior myocardial infarction, prior stroke or transient ischemic attack, congestive heart failure, cancer, chronic obstructive pulmonary disease, cirrhosis, estimated glomerular filtration rate, serum sodium, and hemoglobin.

### 3.2 Predictive performance of stroke volume index for long‐term mortality

The AUC for stroke volume index at predicting mortality in a Cox proportional hazards model was 0.56 at 1, 2, and 3 years. When stroke volume index was combined with 14 clinical covariates (age, body mass index, left ventricular ejection fraction, mean pulmonary artery pressure, systemic systolic blood pressure, prior myocardial infarction, prior stroke/transient ischemic attack, congestive heart failure, cancer, chronic obstructive pulmonary disease, cirrhosis, estimated glomerular filtration rate, serum sodium, and hemoglobin) the AUC was 0.71 at 1 year, 0.71 at 2 years, and 0.70 at 3 years. Model performance was identical when stroke volume index was individually replaced with cardiac index, cardiac power index, stroke work index, and left ventricular work index. Model performance also remained identical when stroke volume index was completely removed from the fully adjusted model (ie, model with 14 clinical covariates without a hemodynamic parameter), with similar discriminatory ability of the model at 1 year (integrated discrimination improvement index = .0021, 95% CI: .0010‐.0034). Little difference in prediction was observed after correcting for optimism (optimism adjusted c‐index [or AUC]). There were no notable violations to the proportional hazards' assumption.

### 3.3 Sensitivity analysis of stroke volume index for long‐term mortality

Landmark analysis was performed on 36 246 patients alive at 30 days. In unadjusted analysis, lower stroke volume index was associated with higher risk of mortality. Compared with the third (middle) quintile of stroke volume index, the risk of mortality was 44% higher in individuals in the lowest quintile and 8% lower in those in the highest quintile of stroke volume index. The association between higher stroke volume index and lower risk of mortality attenuated slightly but remained statistically significant in adjusted analysis such that individuals in the lowest and highest quintile of stroke volume index had 11% higher and 6% lower risk of mortality, respectively, as compared with the participants in third quintile of stroke volume index. In time to event analysis using stroke volume index as a dichotomous variable (low vs high) based on the clinically meaningful cut off of 35 mL/m^2^, low stroke volume index was associated with higher risk of all‐cause mortality in both unadjusted (HR (95% CI): 1.34 (1.29‐1.38), *P* < .001), and adjusted analysis (HR (95% CI): 1.10 (1.06‐1.14), *P* < .001).

The AUC was 0.70 at 1, 2, and 3 years. Model performance was identical when stroke volume index was individually replaced with cardiac index, cardiac power index, stroke work index, and left ventricular work index. Model performance also remained identical when stroke volume index was completely removed from the fully adjusted model (ie, model with 14 clinical covariates without a hemodynamic parameter), with similar discriminatory ability of the model at 1 year (integrated discrimination improvement index = .00075, 95% CI: .00027‐.0013).

## 4 DISCUSSION

In this large observational study, right heart catheterization‐derived stroke volume index was significantly associated with risk of mortality, with a linear association noted between lower stroke volume index and higher risk of mortality. However, stroke volume index and other invasive measures of cardiac performance did not improve the prediction of long‐term mortality independent of clinical covariates. Results were the same in a sensitivity analysis that landmarked subjects who were alive 30 days after their index right heart catheterization.

Low stroke volume and low cardiac power has been associated with high short‐term risk across multiple cardiac and noncardiac conditions including cirrhosis, pulmonary hypertension, severe aortic stenosis, acute heart failure, and cardiogenic shock.^1^, ^3^, ^6^, ^17^, ^18^, ^19^, ^20^, ^21^, ^22^ The present study adds to the existing literature by assessing the long‐term prognostic implications of right heart catheterization‐derived hemodynamic parameters. We observed that low stroke volume index was associated with higher risk of all‐cause mortality. However, stroke volume index did not improve the prediction of mortality when it was added to a list of clinical parameters. This suggests that the low stroke volume index may have limited incremental ability to discriminate between patients who will or will not die on long‐term follow‐up. Thus, routine assessment of the stroke volume index may not add any additional prognostic information above and beyond traditional risk factors for longer‐term outcomes in this patient population.

Our study findings have important clinical implications. Recent studies have demonstrated a decrease in utilization of right heart catheterization among patients with cardiogenic shock with a paradoxical increase in its use among heart failure/valvular heart disease patients without hemodynamic instability, cardiogenic shock, or respiratory failure.^9^, ^10^, ^23^, ^24^ This increase in invasive hemodynamic assessment among less sick patients has been attributed to its greater use among larger, academic hospitals to obtain prognostic data for consideration for advanced therapies.^24^ Furthermore, studies have demonstrated a decline in the risk of short‐term mortality (in‐hospital and 30‐day mortality) among patients undergoing pulmonary artery catheter placement. The decline in short‐term morality among patients undergoing invasive hemodynamic assessment may be reflective of its use in less sick patients or more effective utilization of the hemodynamic data to guide patient care in the short‐term.^9^, ^23^, ^24^ The findings from our study suggest that the invasive measures of stroke volume index or cardiac performance have little prognostic value in the longer‐term, including individuals who survive beyond the first 30‐days after the assessment.

Several factors may underlie the observed findings. First, it is plausible that differences in stroke volume index among lower risk patients (ie, those that survive 30 days after the index right heart catheterization) may not have significant biological relevance, above and beyond the traditional clinical parameters to meaningfully modify long‐term prognosis. Second, it is possible that inaccuracies in measurements may bias toward the null.^25^, ^26^ However, we did observe a significant association between stroke volume index and long‐term outcomes which argues against the role of measurement errors as the key factor underlying the observed prognostic futility of invasively assessed stroke volume.

To the best of our knowledge, this is the largest study which has examined the long‐term prognostic value of right‐heart catheterization hemodynamic parameters. Other strengths of our study include availability of data on important clinical covariates that are controlled for in the adjusted models and availability of mortality up to 3 years of follow‐up. Our study also has some noteworthy limitations. First, considering the unique nature of the patients receiving care at the VA, our findings may not be generalizable to other patient populations, such as women. Second, our findings do not negate the short‐term prognostic value of stroke volume index and other hemodynamic parameters in patients with acute hemodynamic instability, respiratory failure, and cardiogenic shock. Similarly, stroke volume index has been demonstrated to have significant prognostic value in specific patient populations such as those with low‐flow low‐gradient aortic stenosis, cardiac amyloidosis, and heart failure with preserved ejection fraction.

In conclusion, among patients undergoing right heart catheterization at VA hospitals, lower stroke volume index or other measures myocardial performance is modestly associated with higher risk of mortality in longer‐term follow up. However, stroke volume index or other invasive measures of myocardial performance did not add to the prognostic value of risk prediction models for all‐cause mortality above and beyond traditional clinical parameters.

## CONFLICTS OF INTEREST

Dr Anthony A. Bavry discloses receiving honoraria from Edwards Lifesciences and the American College of Cardiology. Dr Dharam J. Kumbhani discloses receiving honoraria from the American College of Cardiology. Dr Stephen Waldo discloses receiving unrelated investigator‐initiated research support from Abiomed, Cardiovascular Systems Incorporated, Janssen Pharmaceuticals, and the National Institutes of Health. Dr Deepak L. Bhatt discloses the following relationships—Advisory Board: Cardax, Cereno Scientific, Elsevier Practice Update Cardiology, Medscape Cardiology, PhaseBio, Regado Biosciences; Board of Directors: Boston VA Research Institute, Society of Cardiovascular Patient Care, TobeSoft; Chair: American Heart Association Quality Oversight Committee; Data Monitoring Committees: Baim Institute for Clinical Research (formerly Harvard Clinical Research Institute, for the PORTICO trial, funded by St. Jude Medical, now Abbott), Cleveland Clinic (including for the ExCEED trial, funded by Edwards), Duke Clinical Research Institute, Mayo Clinic, Mount Sinai School of Medicine (for the ENVISAGE trial, funded by Daiichi Sankyo), Population Health Research Institute; Honoraria: American College of Cardiology (Senior Associate Editor, Clinical Trials and News, ACC.org; Vice‐Chair, ACC Accreditation Committee), Baim Institute for Clinical Research (formerly Harvard Clinical Research Institute; RE‐DUAL PCI clinical trial steering committee funded by Boehringer Ingelheim; AEGIS‐II executive committee funded by CSL Behring), Belvoir Publications (Editor in Chief, Harvard Heart Letter), Duke Clinical Research Institute (clinical trial steering committees, including for the PRONOUNCE trial, funded by Ferring Pharmaceuticals), HMP Global (Editor in Chief, Journal of Invasive Cardiology), Journal of the American College of Cardiology (Guest Editor; Associate Editor), Medtelligence/ReachMD (CME steering committees), Population Health Research Institute (for the COMPASS operations committee, publications committee, steering committee, and USA national co‐leader, funded by Bayer), Slack Publications (Chief Medical Editor, Cardiology Today's Intervention), Society of Cardiovascular Patient Care (Secretary/Treasurer), WebMD (CME steering committees); Other: Clinical Cardiology (Deputy Editor), NCDR‐ACTION Registry Steering Committee (Chair), VA CART Research and Publications Committee (Chair); Research Funding: Abbott, Afimmune, Amarin, Amgen, AstraZeneca, Bayer, Boehringer Ingelheim, Bristol‐Myers Squibb, Chiesi, CSL Behring, Eisai, Ethicon, Ferring Pharmaceuticals, Forest Laboratories, Fractyl, Idorsia, Ironwood, Ischemix, Lilly, Medtronic, PhaseBio, Pfizer, PLx Pharma, Regeneron, Roche, Sanofi Aventis, Synaptic, The Medicines Company; Royalties: Elsevier (Editor, Cardiovascular Intervention: A Companion to Braunwald's Heart Disease); Site Co‐Investigator: Biotronik, Boston Scientific, CSI, St. Jude Medical (now Abbott), Svelte; Trustee: American College of Cardiology; Unfunded Research: FlowCo, Merck, Novo Nordisk, Takeda. No other disclosures were reported.
